# Tardigrade distribution in soils of high Arctic habitats

**DOI:** 10.1002/ece3.11386

**Published:** 2024-07-03

**Authors:** Michala Tůmová, Veronika Jílková, Petr Macek, Miloslav Devetter

**Affiliations:** ^1^ Faculty of Science University of South Bohemia České Budějovice Czech Republic; ^2^ Institute of Soil Biology and Biogeochemistry Biology Centre, Czech Academy of Sciences České Budějovice Czech Republic; ^3^ Institute of Hydrobiology Biology Centre, Czech Academy of Sciences České Budějovice Czech Republic; ^4^ Institute of Agricultural and Environmental Sciences Estonian University of Life Sciences Tartu Estonia

**Keywords:** bird cliff, soil microfauna, soil structure, water bears

## Abstract

Tardigrades are omnipresent microfauna with scarce record on their ecology in soils. Here, we investigated soil inhabiting tardigrade communities in five contrasting polar habitats, evaluating their abundance, diversity, species richness, and species composition. Moreover, we measured selected soil physico‐chemical properties to find the drivers of tardigrade distribution among these habitats. In spite of reported tardigrade viability in extreme conditions, glacier forelands represented a habitat almost devoid of tardigrades. Even dry and wet tundra with soil developing for over more than 10 000 years held low abundances compared to usual numbers of tardigrades in temperate habitats. Polar habitats also differ in species composition, with *Diaforobiotus islandicus* being typical species for dry and *Hypsibius exemplaris* for wet tundra. Overall, tardigrade abundance was affected by the content of nutrients as well as physical properties of soil, i.e. content of total nitrogen (TN), total organic carbon (TOC), stoniness, soil texture and the water holding capacity (WHC). While diversity and species composition were significantly related to soil physical properties such as the bulk density (BD), soil texture, stoniness, and WHC. Physical structure of environment was, therefore, an important predictor of tardigrade distribution in polar habitats. Since many studies failed to identify significant determinants of tardigrade distribution, we encourage scientists to include physical properties of tardigrade habitats as explanatory variables in their studies.

## Graphical abstract

Arctic soil tardigrade communities thrive in habitats with robust plant growth such as dry and wet tundra. Conversely, these communities are virtually absent in glacier forelands and soil crusts. The abundance of tardigrades is intricately connected to soil organic carbon levels, while diversity and species composition are closely associated with soil physical properties such as the BD, texture, stoniness, and water‐holding capacity.

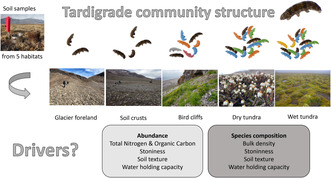

## 1 INTRODUCTION

Tardigrades belong to the microfauna and live in soil, water, and on the surface of plants (Nelson et al., 2015). Tardigrade research in polar regions is relatively abundant. However, it has mostly been focused on cryptogams, mainly mosses (Dastych, 1985; Meininger & Spatt, 1988; Maucci, 1996; Sohlenius & Boström, 2006; Kaczmarek et al., 2012; Zawierucha et al., 2013, 2016, 2017; Zawierucha, Smykla, et al., 2015; Zawierucha, Cytan, et al., 2015; Zawierucha, Podkowa, et al., 2018; Zawierucha, Zmudczyńska‐Skarbek, et al., 2019), and freshwater habitats, mainly cryoconite holes on glaciers (Dastych, 1985; de Smet & van Rompu, 1994; Grøngaard et al., 1999; Zawierucha, Buda, et al., 2018, 2019; Zawierucha et al., 2022). Surveys that included soil samples often investigated also cryptogams, algal or cyanobacterial samples and provided limited information on the difference between the soil and other sample types (Sohlenius et al., 1995, 1995, Courtright et al. 2001, Convey and McInnes 2005, Johansson et al. 2013, Zawierucha et al. 2013). Surveys focused on soil had difficulties to point out conclusions on soil tardigrade distribution due to the high variability of sampled soils (texture, microhabitats, vegetation cover), unbalanced sampling design, and low number of replicates for samples of similar character (Sohlenius et al., 1995, 1995, 2004, Sohlenius and Boström 2005, 2008). Under such circumstances, it was impossible to draw general patterns in species distribution that could be statistically supported.

Tardigrade activity is limited to water‐filled spaces (Nelson et al. 2015), moisture is thus crucial for their active life and reproduction. On the other hand, tardigrades can withstand complete drought entering the dormant state called anhydrobiosis and waiting for moisture for several years (Nkem et al. 2006). On top of that, tardigrade species differ in their ability to enter and survive anhydrobiosis. It seems that their ability to enter anhydrobiosis is connected with the long‐term moisture conditions in the habitat (Horikawa and Higashi 2004). Species mostly found in permanently wet or freshwater environment often show poor anhydrobiotic performance, while species inhabiting periodically drying habitats are among the best anhydrobiotic survivors. Tardigrade species thus show variable preferences for moisture stability rather than momentarilly measured moisture of the substrate. For these reasons, high moisture levels were reported to have a positive, as well as negative or neutral effect on tardigrade abundances (Morgan 1977, Hyvönen and Persson 1996, Briones et al. 1997, Jönsson 2007, Sánchez‐Moreno et al., 2008). Another restraint for tardigrade communities can represent nutrients since they directly impact the amount of tardigrade food sources. Individual tardigrade species differ in their diet and can be classified either as herbi‐fungi‐microbivores feeding predominantly on algae, detritus and fungi or as omni‐carnivores feeding mostly on animal prey (Guidetti et al. 2012). In Svalbard, higher densities of tardigrades were observed in nutrient‐rich areas at well‐developed tundra or nearby seabird colonies, while early successional stages after deglaciation that were strongly limited in nutrients hosted communities with lower densities of individuals (Zawierucha et al. 2016, Devetter et al. 2021). Soil inhabiting tardigrades can be further limited by the physical structure of the soil, which can influence their movement, hunting abilities, and survival of anhydrobiosis (Hohberg and Traunspurger 2005, Poprawa et al. 2022). As an example, Antarctic tardigrades were shown to have species‐specific preferences for certain soil structure (Sohlenius et al. 2004). However, predictors of animal distribution were shown to be often different between Arctic and Antarctic conditions, and organisms might follow different rules in their distribution (Goryachkin et al. 2004, Nielsen and Wall 2013).

Here, we assessed soil tardigrade communities and their distribution in five common Svalbard habitats. Our aim was also to identify the importance of various abiotic factors on their distribution. Since some polar habitats are extremely limited in nutrient content, we expected nutrients to be the main driver of tardigrade distribution. However, as tardigrades seem to depend to a large extent on the physical properties of soil, we also tested if physical properties may shape tardigrade distribution even in a nutrient‐limited environment.

## 2 MATERIALS AND METHODS

### 2.1 Sampling site and habitats description

**FIGURE 1:**
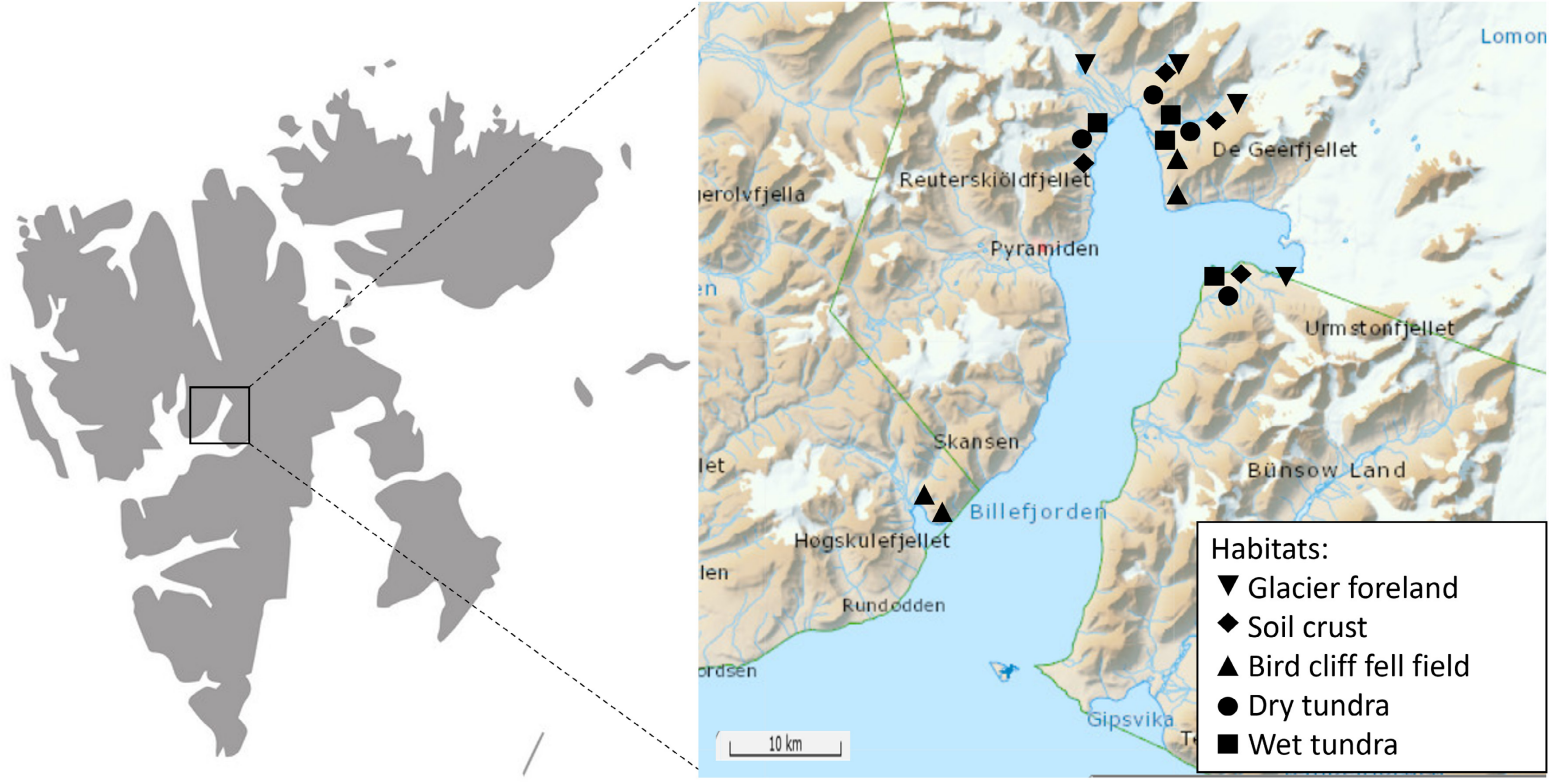
Map of Billefjorden with the 20 investigated locations (4 locations per 5 habitats). Habitats are marked with different symbols (legend provided in the right corner of the figure). With courtesy of the Norwegian Polar Institute.

**FIGURE 2:**
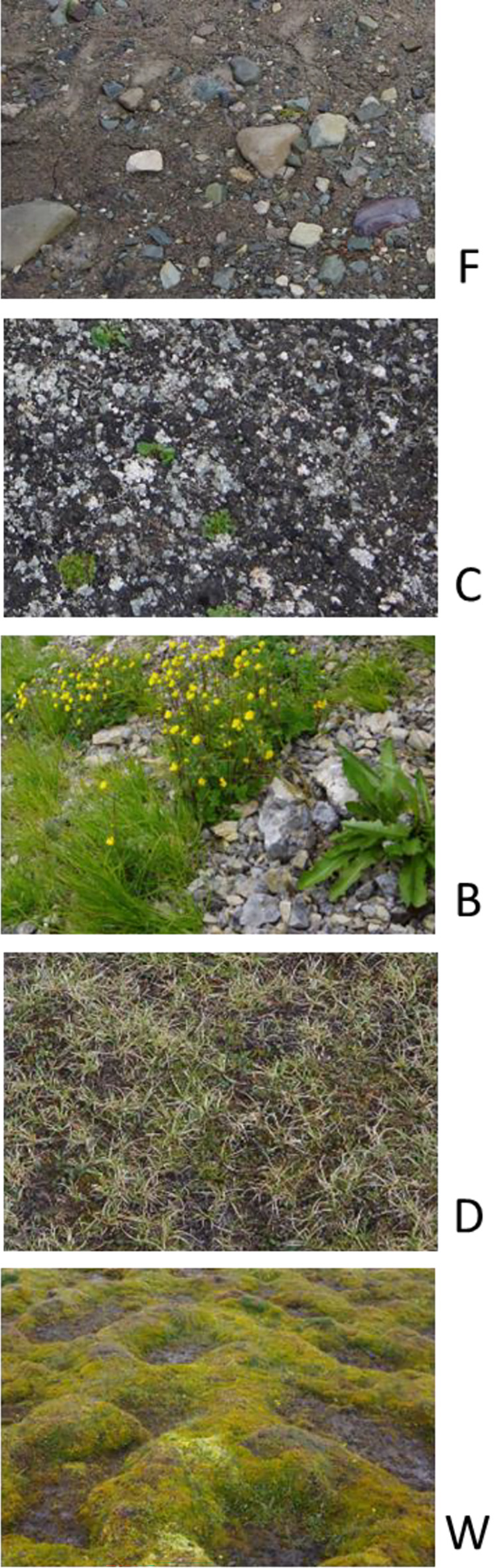
Floor of the investigated habitats. F, Glacier foreland; C, Soil crust; B, Bird cliff fell fields; D, Dry tundra; W, Wet tundra.

**FIGURE 3:**
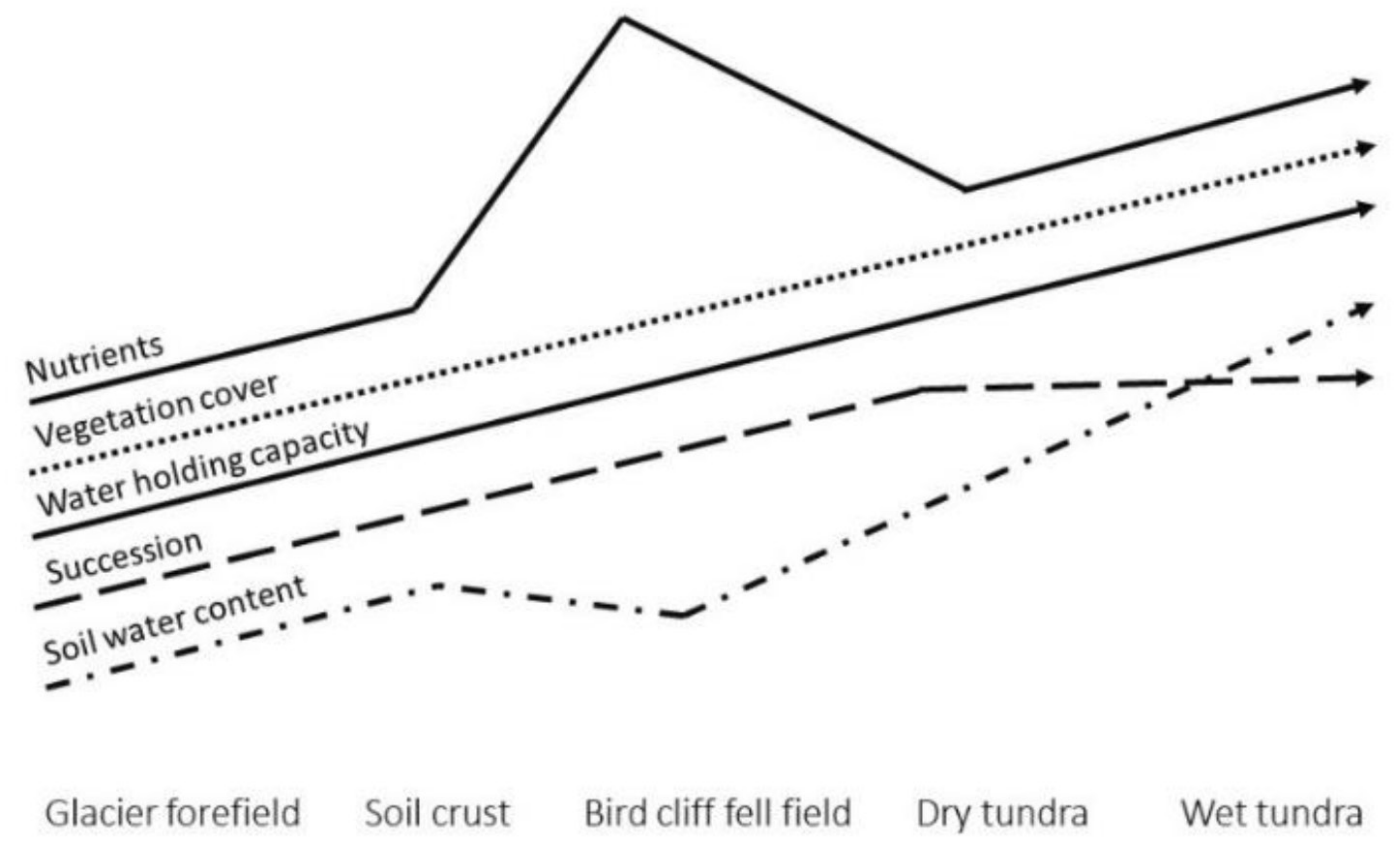
Schematic picture of the gradients of conditions along the investigated habitats. Based on the results of Jílková et al. (2021).

Sampling was performed in Billefjorden in Spitsbergen, Svalbard in 2017 (see the map, Figure 1). We focused on five contrasting habitats that represented a natural gradient of succession, vegetation cover, moisture, nutrients, and soil texture: (1) glacier foreland, (2) soil crust, (3) fell field under bird cliff, (4) dry tundra, and (5) wet tundra (Figures 2 and 3, Jílková et al. 2021).

Glacier foreland represented an initial successional stage almost devoid of vegetation and visibly developed soil crusts. Soils were formed mainly by fine material with high bulk density (BD) and high stoniness (Figure 2). The soil surface was prone to wind erosion and soil movements due to glacial, fluvial, and gravitational processes in the area.

Soil crust represented a young successional stage with a partially stabilized soil surface with terricolous lichens, algae, cyanobacteria, and sparse vegetation (Figure 2).

Contrary to other habitats, the bird cliff fell field was typical by its position on the steep slope. This habitat was characterized by an additional input of nutrients deposited by large seabird colonies (*Fulmarus glacialis*—hundreds of nests) on the cliffs above the investigated slopes, a high richness of vascular plants with high aboveground biomass but intermediate plant cover (Figure 2), and soil consisting of coarse material with considerable stoniness caused by gravel movement. In addition, the bird cliff fell field was characterized by the highest variability of soil conditions even within individual locations. Therefore, location was not included into any of the models as a random factor. Moreover, including location as a random factor resulted in lower AIC of the model.

Dry tundra represented a late stage of successional development. It was characterized by a thin layer of accumulated organic matter which provided nutrients and high coverage of vegetation that consisted of mosses, lichens, and higher plants.

Wet tundra also represented a late stage of successional development but occurred in places with permanently elevated soil moisture. Typical is a formation of hummocks and a higher proportion of mosses (Figure 2). This habitat was typical by the high accumulation of plant biomass and high nutrient content promoted by the frequent occurrence of anseriform birds, which used hummock tundra for pasture and fertilized the soil with excrements.

### 2.2 Sample collection and processing

Soil cores for soil tardigrade extraction were sampled with a cylindrical metal corer (5.5 cm diameter, 4 cm height). All soil samples were taken without vegetation admixtures—all green parts were removed. The cores were gently homogenized by hand before extraction of tardigrades, and 50 mL of soil was weighed and, tardigrades were extracted by high‐gradient Baermann funnels (Czerneková et al. 2018). Extracted tardigrade individuals were counted and preserved on permanent slides in Hoyer's medium. The species were identified according to the original literature (for full annotation, see Supplementary Material S1). Some of the individuals were not identified (9%) due to distortions inflicted during fixation or bad visibility of the taxonomically informative structures. Moreover, some of the individuals were determined only to genus level, because the determination of species required the presence of the eggs that we failed to extract or more individuals for assessment of morphometric differences. Samples for physico‐chemical soil properties, i.e. stoniness, BD, soil water content (SWC), water‐holding capacity (WHC), total organic carbon (TOC), total nitrogen (TN), and soil texture were collected and analyzed as described in Jílková et al. (2021) and they are summarized in Tables 1 and 2.

**TABLE 1:** Mean values of stoniness, bulk density (BD), soil water content (SWC), water‐holding capacity (WHC), the content of total organic carbon (TOC), and total nitrogen (TN) in the observed habitats.

Habitat	Stoniness (%)	BD (g cm^−3^)	SWC (%)	WHC (%)	TOC (mg g^−1^)	TN (mg g^−1^)
Foreland	31.5 ± 3.6	1.0 ± 0.0	9.3 ± 0.8	21.8 ± 1.3	3.2 ± 0.5	0.3 ± 0.0
Soil crust	18.9 ± 2.8	1.1 ± 0.1	22.0 ± 1.1	32.7 ± 2.2	26.8 ± 2.2	2.2 ± 0.2
Bird cliff	69.6 ± 2.8	0.2 ± 0.0	18.0 ± 2.6	57.6 ± 8.4	74.6 ± 8.3	5.7 ± 0.6
Dry tundra	4.2 ± 2.2	0.8 ± 0.0	30.7 ± 2.5	70.2 ± 5.5	55.6 ± 7.1	4.1 ± 0.7
Wet tundra	2.0 ± 1.8	0.7 ± 0.3	49.4 ± 4.2	124.0 ± 22.8	57.0 ± 12.4	4.1 ± 1.0

*Note*: Number of replicates per habitat *n* = 4. Modified from Jílková et al. (2021).

**TABLE 2:** Mineral particle size distribution (%) in the studied habitats.

Habitat	Coarse sand 2000–630 μm	Medium sand 630–200 μm	Fine sand 200–63 μm	Coarse silt 63–20 μm	Medium silt 20–6,3 μm	Fine silt 6,3–2 μm	Clay <2 μm
Foreland	11.3 ± 2.2	12.7 ± 1.8	23.5 ± 4.8	21.5 ± 4.2	13.1 ± 2.9	4.8 ± 0.8	13.3 ± 3.5
Soil crust	5.0 ± 1.7	9.5 ± 2.0	29.7 ± 5.2	22.9 ± 5.1	11.8 ± 2.3	4.5 ± 2.3	14.3 ± 2.1
Bird cliff	28.0 ± 4.2	12.7 ± 3.6	12.4 ± 2.3	10.6 ± 2.8	9.3 ± 2.0	5.1 ± 1.2	19.8 ± 2.3
Dry tundra	1.6 ± 0.3	10.4 ± 2.3	33.8 ± 8.3	26.6 ± 6.2	9.0 ± 2.7	3.8 ± 2.7	12.6 ± 3.5
Wet tundra	1.6 ± 0.6	8.5 ± 2.5	35.1 ± 7.8	27.7 ± 5.6	5.6 ± 1.0	8.1 ± 5.3	13.5 ± 3.6

*Note*: Values are means ± SE. Modified from Jílková et al. (2021).

All samples were obtained during July 2017, covering the first half of the growing season (Karlsen et al. 2022). Each type of habitat was sampled at 4 different locations and each of these locations was sampled in 5 randomly chosen replicate plots. A plot was designed as a 0.5 × 0.5 m^2^. In total, 100 samples were collected (5 habitats × 4 locations × 5 replicates per location). Samples for the measurement of physico‐chemical properties were taken from the same square plots as the samples for tardigrade extraction (total number of samples = 100). The only exception was soil texture, which was sampled in one replicate per location due to time‐consuming sample processing (total number of samples = 20).

### 2.3 Statistical analyses

In all statistical tests, data of dependent as well as independent variables were averaged into one value per location. This way, each location represented one independent measurement for the analyses.

The differences in overall abundance, species richness, and Shannon index among individual habitats were evaluated using one‐way ANOVAs with subsequent post hoc tests with Tukey contrasts. To evaluate the possible effect of location on the abundance, species richness and the Shannon index, we used full dataset (100 observations) to model location as a random factor. However, models that included location as random factor resulted in lower AIC of a model. The differences among habitats thus cannot be explained by the differences among localities.

We also tested the effect of physico‐chemical soil properties on abundance, species richness, and the Shannon index using simple regressions. Significant predictor variables were fitted with polynomial curves (stoniness, coarse sand content) or linear lines (WHC, TOC, and TN) depending on their relationship with response variables. In order to identify the best predictors for our response variables we ran AIC stepwise selection using only those physico‐chemical properties that resulted as significant in simple regression. R software (R Core Team 2017) was used for analyses computation and figures construction. Data for abundance were logarithmically transformed to increase the normality of data in both tests.

Species composition and their relationship to, separately, habitat and physico‐chemical properties were evaluated by constrained ordination analyses, Redundancy Analyses (RDA), using the software CANOCO 5 (ter Braak and Šmilauer 2012). Species compositions were evaluated as total number of individuals per each species. The response data were logarithmically transformed to decrease the weight of the dominant species. Results are presented as an adjusted explained variation (which controls for the number of explanatory variables). The significance of the model for individual species was tested by *t*‐value biplots (Van Dobben circles).

## 3 RESULTS

Of 100 samples, 52 contained tardigrades. Specifically, 167 individuals that belonged to 18 taxa were extracted (Table 3).

**TABLE 3:** List of recorded tardigrade species, with the total number of species, individuals and positive samples found within each habitat.

Taxon	Glacier foreland [ind]	Soil crust [ind]	Bird cliff [ind]	Dry tundra [ind]	Wet tundra [ind]
*Milnesium* sp.				3	
*Hypsibius* cf. *convergens*		1			
*Hypsibius microps*	2			8	
*Hypsibius exemplaris*		2		1	6
*Fractonotus* sp.				1	
*Ramazzottius* sp.		2	2	6	4
*Isohypsibius coulsoni*				1	
*Ursulinius elegans*	1	2	3	1	
*Bertolanius nebulosus*					1
*Diphascon pingue* group			1	10	10
*Diphascon nobilei* group			12	1	1
*Guidettion* cf. *modestum*				7	
*Adropion belgicae*				5	
*Adropion scoticum*		1		1	2
*Platicrista angustata*					3
*Mesobiotus* sp.			1		1
*Paramacrobiotus richtersi* group					16
*Diaforobiotus islandicus*			6	21	3
Total number of species	2	5	6	13	10
Total number of individuals	3	8	25	66	47
Total number of positive samples	2	9	9	16	16

*Note*: Total number of samples per habitat, *n* = 20 (4 localities × 5 samples, 1 sample = 50 mL of soil).

### 3.1 Tardigrade abundance and species richness

**FIGURE 4:**
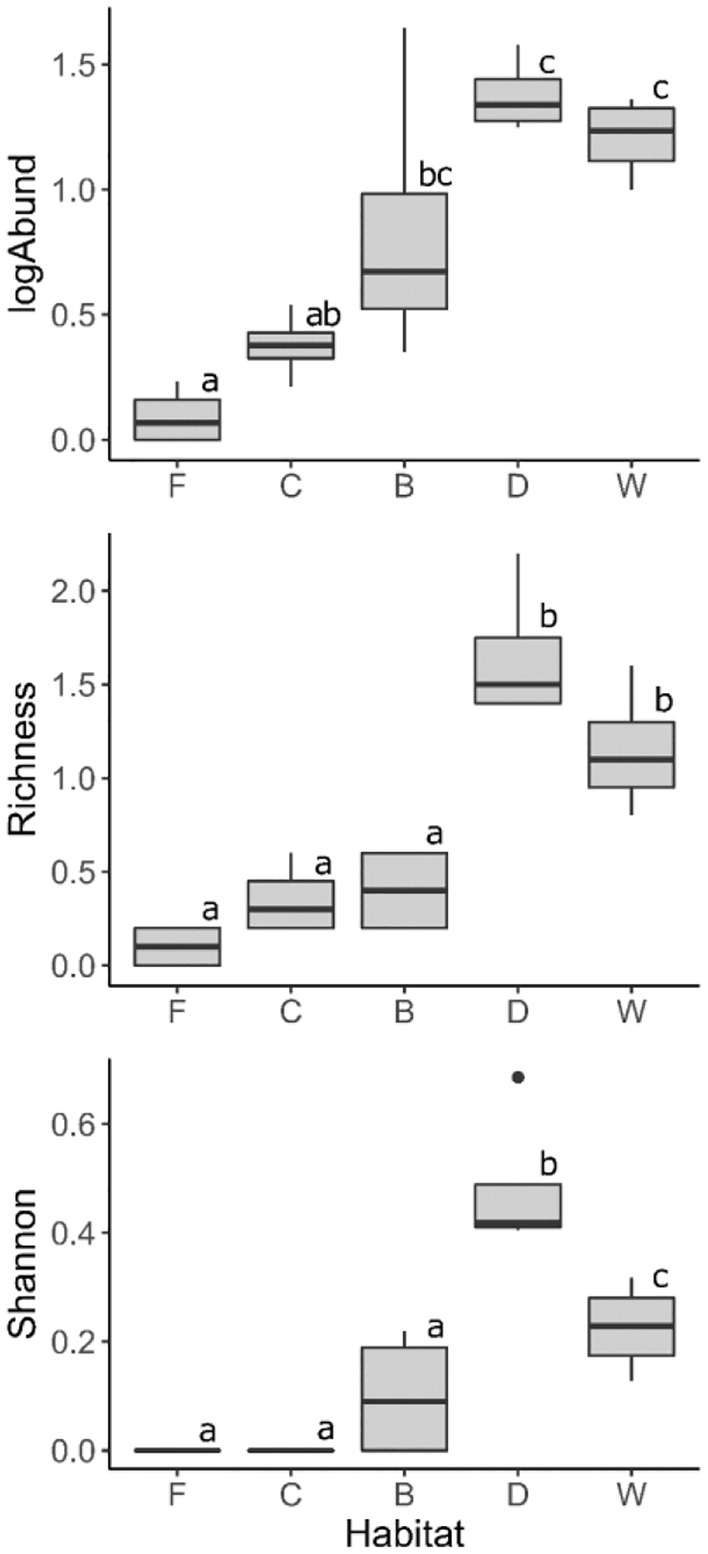
Abundance, species richness, and Shannon index of soil tardigrade communities in five habitats (F, glacier foreland; C, soil crust; B, bird cliff fell field; D, dry tundra; W, wet tundra). For each habitat we have four replicates (calculated as average number from 20 samples; 4 locations × 5 samples taken at each location). Medians are represented by black lines in the middle of the boxes, box represent 25%–75% quartile of all observations, whiskers lower and upper quartiles and points represent outliers. Habitats that do not have a letter in common differ significantly.

Tardigrades were extremely rare in soils of glacier foreland (Figure 4). Only two samples out of 20 contained tardigrades. Three individuals of the two species were found. Soil crust samples contained tardigrades in 45% of cases and 5 tardigrade species were identified. With more than half of the samples without tardigrades, the mean abundance in soil crust samples remained rather low (0.015 ind. g^−1^ dw; 2003 ind. m^−2^). Dry and wet tundra contained the most abundant and species‐rich communities (Figure 4). Dry tundra rendered 13 species and an average abundance of 0.24 ind. g^−1^ dw; 18,190 ind. m^−2^ and samples from wet tundra provided 10 species and an average abundance of 0.16 ind. g^−1^ dw; 13,096 ind. m^−2^. At the same time, dry tundra provided the highest value of the Shannon index, indicating the highest diversity (Figure 4). Bird cliff fell field had an intermediate abundance of 0.13 ind. g^−1^ dw; 7886 ind. m^−2^ alongside with 6 species. However, samples taken in the bird cliff fell field were characteristic by the most variable abundance, ranging from 0.02–2.1 ind. g^−1^ dw or 875–24,831 ind. m^−2^.

### 3.2 Tardigrade species composition

**FIGURE 5:**
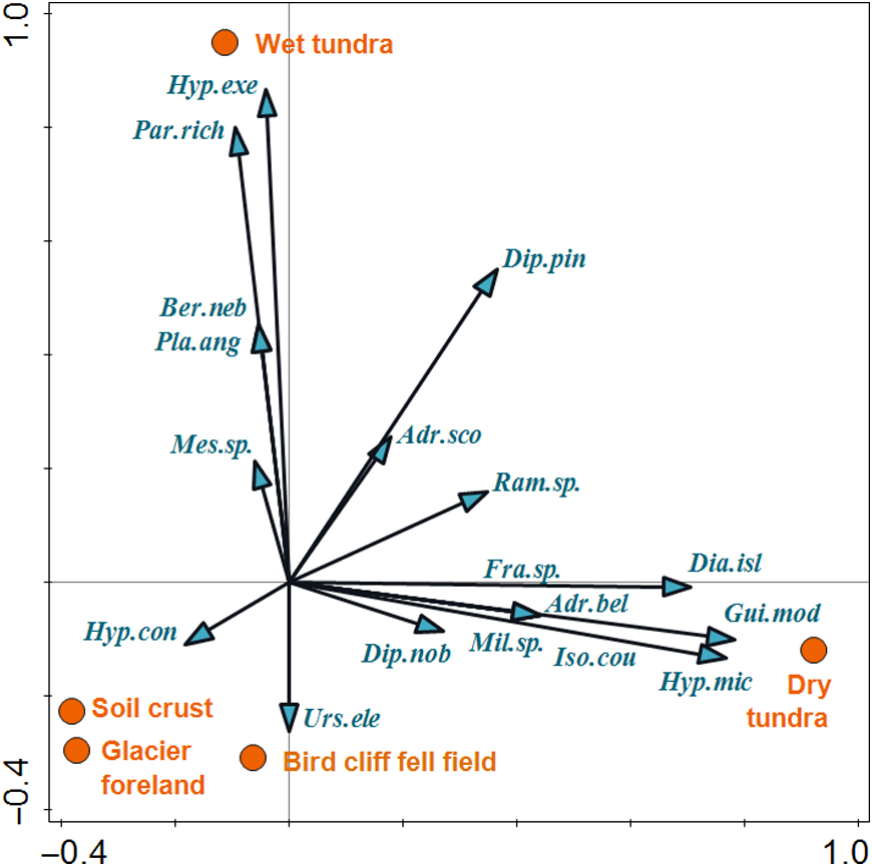
Ordination diagram (Constrained RDA) showing the difference in species composition among the habitats. Abbreviations of the species are based on the first three letters of the genus name and species name.

Type of habitat explained 26.4% of the variability in the tardigrade species composition (RDA, test on all constrained axes: *F* = 2.7, *p* = .002; Figure 5). Most of the differences were explained by the composition of species in dry and wet tundra since 77% of the tardigrades´ individuals were observed in the samples from these two habitats. Therefore, the first ordination axis depicted differences between dry tundra on one side and glacier foreland, soil crust and bird cliff fell field on the other side, while the second ordination axis depicted the difference between wet tundra and the other habitats (Figure 5). In addition, all species found in samples from glacier foreland, soil crust, and bird cliff fell field were also found in at least one of the two habitats of higher successional stage (Table 3). One exception was *Hypsibius* cf. *convergens*, which was present as one individual in a sample from the soil crust. In contrast, dry and wet tundra communities held some species that preferred one of these habitats. Specifically, most individuals of *Diaforobiotus islandicus* were recorded in dry tundra. *Hypsibius microps* was missing in wet tundra having high number of records from dry tundra, and *Guidettion* cf. *modestum* was present exclusively in dry tundra. In contrast, *Hypsibius exemplaris* had higher abundance in wet tundra compared to dry tundra, while *Paramacrobiotus* aff. *richtersi* was observed exclusively in wet tundra. Furthermore, other species occurred in only one habitat but in low abundances, not allowing to retrieve a statistically significant relationship to the habitat. Of these, *Milnesium* sp., *Adropion belgicae*, *Fractonotus* sp., and *Isohypsibius coulsoni* were found only in dry tundra samples, while *Platicrista angustata* and *Bertolanius nebulosus* were found only in wet tundra samples.

### 3.3 Effect of soil properties on tardigrade abundance

**FIGURE 6:**
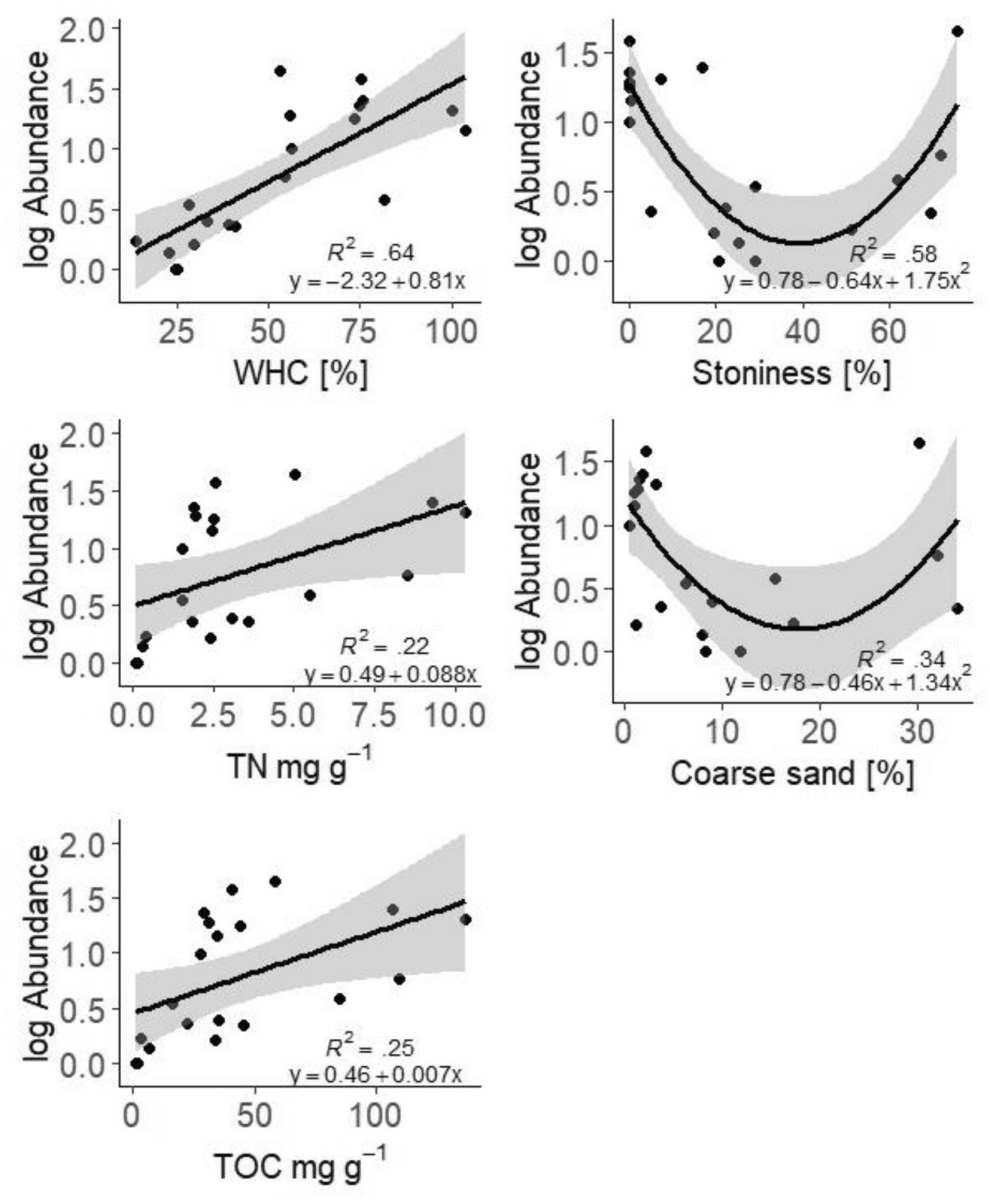
Simple regression of abundance and water‐holding capacity (WHC), total content of organic carbon (TOC), total nitrogen (TN), stoniness and coarse sand content (fraction of soil particles 2000–630 μm in size).

In simple regression, abundance showed a positive linear correlation with water‐holding capacity (WHC) (*R* ^2^ = .64, *p* < .001), TOC (*R* ^2^ = .25, *p* = .023, Figure 6), and TN (*R* ^2^ = .22, *p* = .035, Figure 6). Abundance also showed a quadratic relationship with stoniness and coarse sand content (*R* ^2^ = .58, *p* < .001 and *R* ^2^ = .34, *p* < .05, respectively). As can be seen in Figure 6, high abundances were observed in soils either with very low or very high content of stones and coarse sand. Based on the AIC stepwise selection, only stoniness and TN were added to the final model explaining 68% of the variability (*F*_3,16_ = 14.38, *p* < .001).

### 3.4 Effect of soil properties on tardigrade species richness, Shannon index, and species composition

**FIGURE 7:**
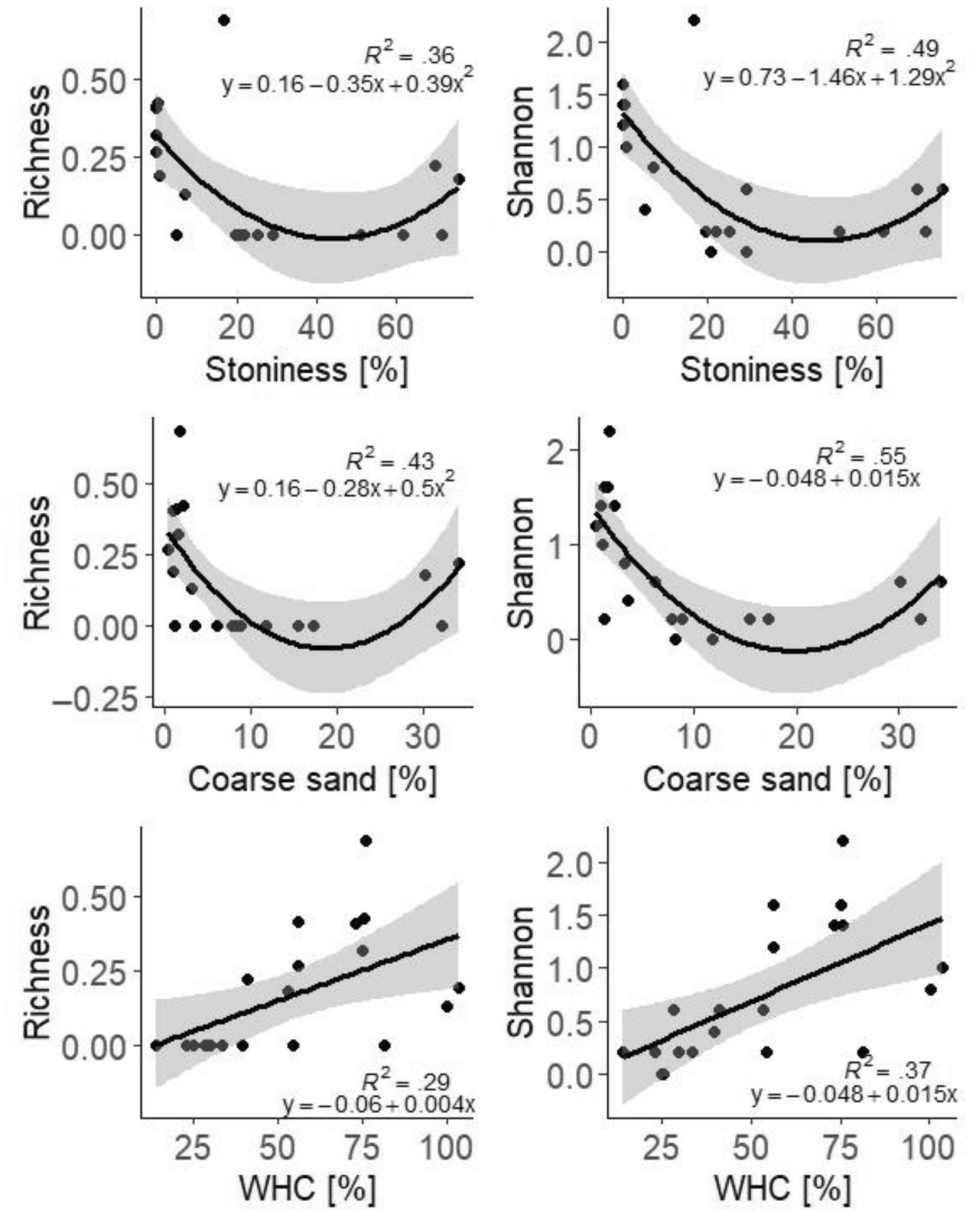
Simple regression of species richness and Shannon index with stoniness, coarse sand content (fraction of soil particles 2000–630 μm in size) and water‐holding capacity (WHC).

Species richness and Shannon index had quadratic or linear relationships with several soil physical properties (Figure 7). Specifically, both followed the same pattern as the abundance when related to WHC (*R* ^2^ = .29, *p* < .01 and *R* ^2^ = .37, *p* < .01, for species richness and Shannon index, respectively), stoniness (*R* ^2^ = .36, *p* < .05 and *R* ^2^ = .49, *p* < .01, respectively) and coarse sand content (*R* ^2^ = .43, *p* < .01 and *R* ^2^ = .55, *p* < .01, respectively). In the final model based on AIC stepwise selection, species richness as well as Shannon index were explained by coarse sand content and WHC (*R* ^2^ = .58, *F*_4,15_ = 7.62, *p* = .001 and *R* ^2^ = .56, *F*_4,15_ = 4.71, *p* = .01, respectively).

**FIGURE 8:**
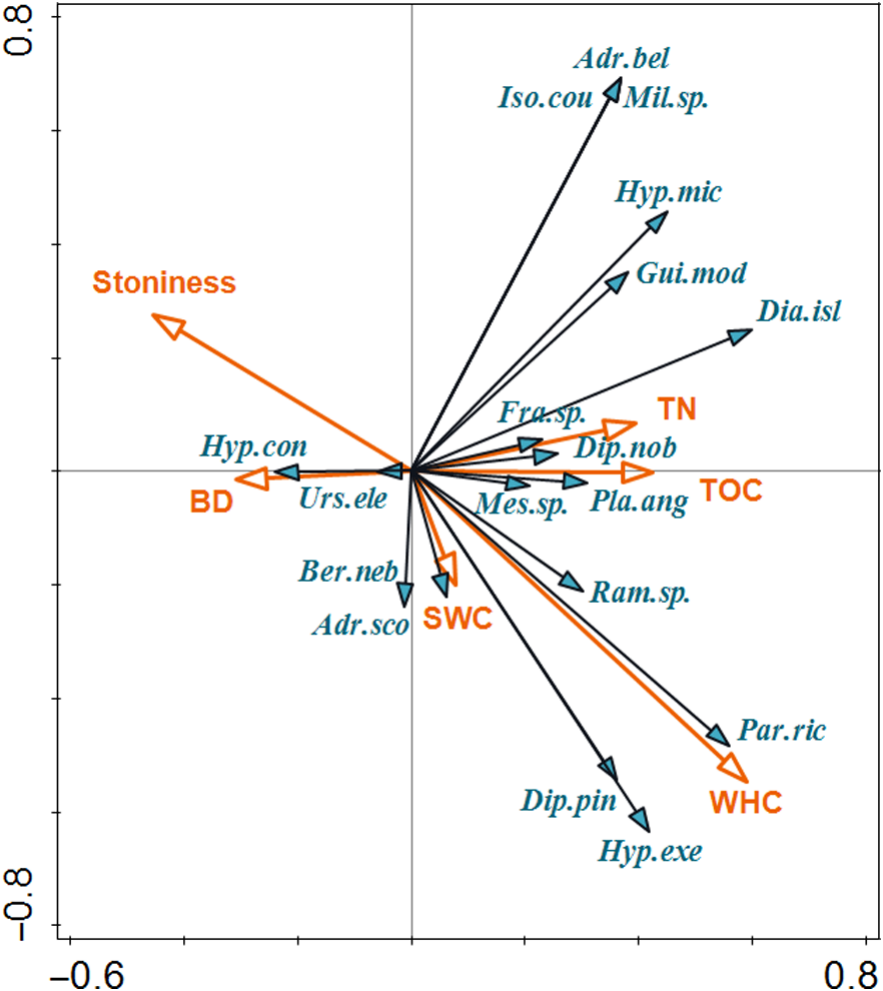
Ordination diagram (Constrained RDA) showing correlation of species with physico‐chemical soil properties. BD, bulk density; SWC, soil water content; TN, total nitrogen; TOC, total organic carbon; WHC, water‐holding capacity. Abbreviations of the species are based on the first three letters of the genus name and species name.

Soil physico‐chemical properties explained 26.7% of the variability in the tardigrade species composition (RDA, test on all constrained axes: *F* = 2.2, *p* = .006; Figure 8). Explained variation was covered by BD (explained 12%, *p* = .028). Neither of the other variables significantly contributed to the explained variation.

## 4 DISCUSSION

In general, the abundance of soil tardigrades in polar regions seems to be low. Sohlenius et al. (1995, 1995) reported a mean abundance of 2 ind. g^−1^ dw of soil in Antarctica. Our results showed even much lower mean abundances, i.e. less than 1 ind. g^−1^ dw. Other studies from polar regions recorded higher tardigrade numbers, i.e. 6 ind. g^−1^ dw (Zawierucha et al. 2013), 9 ind. g^−1^ dw (Zawierucha et al. 2016), and 12 ind. g^−1^ dw (Sohlenius and Boström 2005). However, soil in these studies was mostly an admixture of very light moss samples, and mosses usually host higher densities of tardigrades than soil samples (Sohlenius and Boström 2008, Zawierucha et al. 2017). In the current study, we carefully removed all green parts of plants and mosses from the samples which probably resulted in lower abundances.

Only three individual tardigrades were found in glacier forelands, implying that tardigrades do not inhabit this environment and the found specimens were probably transported there by wind (Nkem et al. 2006). Previously, scarcity or even lack of microfauna including tardigrades have been recorded in extreme environments of polar deserts or nunataks of Antarctica (Sohlenius et al., 1995, 1995, Courtright et al. 2001). Concordantly, soil tardigrades have already been reported to have low abundances not only tens but also hundreds of years after deglaciation at central Spitzbergen (Devetter et al. 2021). However, less developed habitats in the temperate zone, such as early successional stages in inland sand dunes and in mines, supported much higher abundances than older stages that were up to 100 years old, i.e. up to 10^5^ ind. m^−2^ (Russell et al. 1994, Hohberg 2006, Frouz et al. 2008, Hohberg et al. 2011). The observed discrepancy between the polar and temperate zone may stem from the fact that all processes, including succession, are very slow in the Arctic due to low temperatures and short vegetation season usually lasting 60 days (Karlsen et al. 2022). However, the results seem to depend on the age of habitats under comparison. The ecosystems with long‐term uninterrupted development, such as broad‐leaved forests in Spain, have been reported to harbor high abundances and diversity of tardigrades (Guil & Sánchez‐Moreno, 2013). This is in accordance with our results, where dry tundra that develops for more than 10,000 years in the studied area (Devetter et al. 2021) rendered the highest abundance, species richness, and diversity.

We observed that the factors underlying differences in tardigrade communities were a combination of soil physical properties and nutrients. In the current study, nutrients were an important driver for the overall abundance of tardigrades. Contrastingly, in the previous manipulative experiments, tardigrade abundance was correlated with mineral nitrogen neither after the addition of nitrogen fertilizer nor before the manipulation (Hyvönen and Persson 1996, Sánchez‐Moreno et al., 2008). However, polar habitats are often nutrient‐limited (Devetter et al. 2021, Jílková et al. 2021) and the observed differences in nutrient content among polar habitats can show more pronounced effects on tardigrades than in the previous studies, i.e. in coniferous soils of central Sweden (Hyvönen and Persson 1996) and oak, laurel and pine forests of California (Sánchez‐Moreno et al., 2008).

Abundance was also related to the physical properties of soil, based on the AIC stepwise selection, mainly to stoniness that probably reflected the stability of the environment. High stoniness in glacier foreland, soil crust, and bird cliff fell fields was caused by the short development of soil and soil movements caused by glacial, gravitational, and fluvial processes. Therefore, abundance was lower at places with high instability. However, bird cliff fell fields had low stability and high nutrient content at the same time and harbored high abundances of tardigrades despite high stoniness and instability. As a result, abundance had a quadratic relationship to stoniness and coarse sand content, because it was high at tundral habitats with very low stoniness and high nutrient content and at bird cliff fell fields with very high stoniness and even higher nutrient content.

Two localities of bird cliff fell fields were located at the considerable distance from the others. To exclude the effect of location on the results, we compared AIC of the models with and without location modeled as a random factor before we performed the analyses presented in this article. Based on the lower AIC value of the model without location, the effect of location was not significant.

Not only abundance but also the Shannon index and species richness correlated with soil physical properties. They were best explained by coarse sand content and WHC. Soil that holds water for longer periods of time (high WHC) seems to support more species than places that undergo drying more often. The effect of WHC and no concurrent effect of SWC shows that hydration capacity was a more important factor than actual moisture for tardigrade species richness and diversity. The reason may be that some tardigrade species are susceptible to quick drying and require a slow transition to anhydrobiosis. Meaning that substrates maintaining a higher amount of water provides more time for active life and more time for drying (Wright 1991).

Species composition analysis showed higher abundances of some species either in dry or wet tundra. *Diaforobiotus islandicus* which held high abundances in dry tundra had already been ascertained as a tundral signature species (Dastych 1985, Maucci 1996). Similarly, *Hypsibius exemplaris*, that had higher abundance in wet tundra, had previously been recorded as a species that frequently occurs in moist habitats (Gasiorek et al., 2018). We also documented exclusive presence of the *Paramacrobiotus richtersi* group in wet tundra and high numbers of *Hypsibius microps* in dry tundra, but no inclination for moisture level had been reported for these species before. In the further analysis (species composition vs. abiotic factors as explaining variables), most variability was explained by the BD (conditional effect, explained 12.6%, *p* = .012), not the variables connected to moisture content (the WHC or SWC). Therefore, our results suggest that other differences in soil physical properties besides moisture distinguishing wet and dry tundra could underly species preferences for one of these habitats.

Soil physical structure interferes with many aspects of tardigrade life. Tardigrades cannot dig in the soil, their movement, therefore, relies on the existing soil structure. Inconvenient soil structure may decrease tardigrade hunting efficiency (Hohberg and Traunspurger 2005) and anhydrobiotic survival (Poprawa et al. 2022). However, a causal relationship between tardigrades and physical soil properties implied by our results can be confirmed only by manipulative experiments.

This study as many others struggles with high variability of tardigrade distribution and although we sampled as many samples as we were able to process, we still collected only 167 individuals, which is a low number to draw general patterns of their distribution. However, our data point out an interesting implication that soil structure may play an important role in tardigrade distribution in polar regions and draw possible directions for future research on soil‐inhabiting tardigrades.

## AUTHOR CONTRIBUTIONS

**Michala Tůmová:** Data curation (lead); investigation (equal); methodology (supporting); visualization (lead); writing – original draft (lead); writing – review and editing (equal). **Veronika Jílková:** Data curation (supporting); investigation (equal); methodology (equal); writing – review and editing (equal). **Petr Macek:** Conceptualization (lead); funding acquisition (lead); investigation (equal); methodology (equal); project administration (lead); writing – review and editing (equal). **Miloslav Devetter:** Conceptualization (supporting); funding acquisition (supporting); investigation (equal); methodology (equal); project administration (supporting); supervision (lead); writing – review and editing (equal).

## CONFLICT OF INTEREST STATEMENT

We have no conflicts of interest to declare.

## Supporting information


Supplementary Material S1.

Supplementary material S2.

## Data Availability

The data that supports the findings of this study are available in Supplementary Material S2 of this article.
