# Supplementary material for: Tardigrade distribution in soils of high Arctic habitats
Source: Ecol Evol. 2024 Jul 3;14(7):e11386. doi: 10.1002/ece3.11386 (PMC11222015; doi:10.1002/ece3.11386)
Supplement: Supplementary file 1 — Supplementary Material S1. [file ECE3-14-e11386-s002.docx]

**Supporting information 1**

**Annotated list of observed species**

Here, we provide an overview of taxonomic traits used for the identification of the extracted tardigrades. In some taxa, we observed slight differences in appearance from those provided in the original literature. Discrepancies between original descriptions and our observations provide an idea of the natural variation of some traits, point out some overlooked traits that can help with their identification, and facilitate the future update of the taxonomical identity of observed species. All the traits have been observed after fixation in Hoyer´s medium using a light microscope with phase or Nomarski Differential Interference Contrast up to the magnification of 400x. Only information on traits that are currently important for the identification of species and differences with original descriptions are described. For identification, we followed the original literature with species descriptions listed in the 41^st^ edition of the Actual checklist of Tardigrada species (Degma & Guidetti, 2009-2022) and selected literature that contained determination keys for specific groups of tardigrades.

***Milnesium* sp**. (n=3) Observed individuals had smooth cuticles, tiny accessory points on primary branches of external claws, and massive cuticular bars under the claws of the first three pairs of legs. Since some species can be distinguished based only on statistical morphometry or DNA sequence (Morek et al., 2019), we decided to determine this taxon only at the genus level.

***Hypsibius* cf. *convergens*** (n=1) The only observed individual had a body size of 149 µm, sub ventral mouth, two macroplacoids, and no microplacoid or septulum in the pharynx. Macroplacoids had the form of rounded rods and measured 4.0, and 3.0 µm, respectively. The first macroplacoid was constricted in the middle. Buccal tube width was 1.5 µm (internal), and 2.6 µm (external). Claws lacked lunulae and cuticular bars. Claws on the first pair of legs measured 8.0 µm (external), and 6.5 µm (internal); on the fourth pair of legs 10.6 µm (posterior), 7.0 µm (anterior). The species cannot be determined with certainty, because the original description of the species is incomplete (Gasiorek et al., 2018).

***Hypsibius microps*** (n=10) Observed individuals had smooth cuticles, a slightly oval pharynx with a length/width ratio of 1.2, two granular macroplacoids, and no microplacoid or septulum. The first macroplacoid was longer than the second. Claws had no lunulae. Accessory points were present on the main branches of all claws. There were no cuticular bars on any of the legs. This species was distinguished from other members of convergens group by granular macroplacoids without constrictions, which is a trait shared with *Hypsibius pallidus* (Kaczmarek & Michalczyk, 2009). From *H. pallidus* it differed by the following morphometric traits: mean body size 185 µm, buccal tube 0.7 - 1 µm (internal), 1.32 - 1.9 µm (external), mean length of the first macroplacoid 2.5 µm, mean length of the second macroplacoid 2.0 µm (Kaczmarek & Michalczyk, 2009).

***Hypsibius exemplaris*** (n=9) Observed individuals had smooth cuticles, a mean body size of 187.4 µm, a mean buccal tube width of 1.5 µm (internal), 2.3 µm (external), and small pharynx with length/width ratio 1.35 that contained two macroplacoids and a septulum. Macroplacoids had the form of rounded rods and mean lengths of 4.1 µm, and 3.4 µm; respectively. The mean length of the septulum was 1.8 µm. From other similar species, it differed by the arrangement of cuticular bars near claws, the size of the claws, and the point of stylet support insertion (Gasiorek et al., 2018). The first three pairs of legs were without cuticular bars. On the fourth pair of legs, there were cuticular bars between the anterior and posterior claws, connected to the base of the posterior claw. The length of claws on the fourth pair of legs was on average 12.2 µm (posterior), and 8.25 µm (anterior). Stylet supports were inserted on average at 67% of the buccal tube length.

***Fractonotus* sp.** (n=1) The only found individual had claws and furcae corresponding best with the genus *Fractonotus* (Gasiorek et al., 2019), however, we could not spot the elliptical organs in the head and the AISM was not well visible. The tardigrade had a small body size of 164 µm, irregular dense small tubercles on the cuticle, sub-terminal mouth, buccal tube width of 1 µm (internal), 1.8 µm (external), large pharyngeal apophysis, spherical pharynx with two granular macroplacoids and no microplacoid or septulum. The first macroplacoid was longer (3.1 µm) than the second (2.3 µm) and strongly constricted. The claws were small. Their position was not optimal for measurements, but the size of the external and posterior claws is about 8 µm, and the internal and anterior claws are about 5 µm. Claws had a thin triangular basal part, and no lunules or cuticular bars. Main branches bore accessory points.

***Ramazzottius* sp.** (n=14) Our individuals had a mean body size of 220 µm, subterminal mouth, buccal tube width of 0.7 – 1.6 µm (internal), 1.6 - 2.7 µm (external), pharynx length/width ratio 1.0 - 1.3, two granular macroplacoids and sometimes tiny microplacoid. The first macroplacoid was a bit longer than the second, but the difference in macroplacoid size was small. The mean length of the macroplacoids was 3 µm and 2.4 µm, respectively. Claws had accessory points on all main branches, no lunulae, and no cuticular bars. Posterior claws on the fourth pair of legs had always a lower pt ratio than 100. Our individuals were determined to be the genus *Ramazzottius* based on the morphology of claws (*Ramazzottius*-type) and by the lack of flexible part of the pharyngeal tube (which is present in *Hebesuncus*), the lack of dark-pigmentation, cuticular bars on the legs and the presence of accessory points (compared to *Cryoconicus*). The individuals were determined at the genus level because many characteristics overlap among species and some of the species are possible to discern only based on the egg morphology (Biserov, 1997-98).

***Isohypsibius coulsoni* (n=1)** The only individual was positioned on its back, thus we could observe only the ventral part of the body. The individual had a smooth cuticle on the ventral side, eye spots, a terminal mouth, and a body size of 321 µm. The oral cavity armature was not visible. Buccal tube width measured 2 µm (internal), and 4 µm (external). Pharynx was slightly oval 29 x 23 µm and contained large apophyses, three macroplacoids, and a microplacoid. Macroplacoids were oval rounded rods of almost equal length (4.2, 4.0, and 4.2 µm; respectively). Microplacoid measured 2 µm. The main branches of the claws had accessory points. Cuticular bars were present under the claws of the first three pairs of legs. Claws on the fourth pair of legs measured 20.6 µm (posterior), and 12.7 µm (anterior) (Kaczmarek et al., 2012).

***Ursulinius elegans* (n=7)** At the first sight, the individuals were slightly brownish and had large eyes and folds on the cuticle. Gibbosities and polygonal mesh were not visible. Under magnification 400x, tiny granulation in most of the individuals had been observed, especially in the rear part of the body. Individuals had sub ventral mouths, buccal tube widths around 2 µm (internal), 4 µm (external), and slightly oval pharynges with a length/width ratio of 1.1. The pharynx contained large apophyses, two often constricted macroplacoids, and a microplacoid. Macroplacoids measured 6.7 and 4.2 µm; respectively. Claws had lunulae and accessory points on the main branches. Long, double-layered, and wavy cuticular bars were present on the first three pairs of legs. External claws measured I 17-20 (pt 48 - 62), II 18 µm (pt 51), III 17-19 µm (pt 0.48), IV 19-23 (pt 51-58).

***Bertolanius nebulosus*** (n=1) The only individual had a body with a length of 416 µm, mouth surrounded by lamellae (probably 14), buccal tube width of 4.71 µm (internal), 6.79 µm (external), pharynx with two macroplacoids and no microplacoid or septulum. In the first third of the first macroplacoid there was a protrusion and just after it also an incision. The second macroplacoid had a protrusion caudally. Macroplacoids lengths were 15.0, and 11.0 µm; respectively. Claws were of *Eohypsibius*-type with accessory points on main branches. Following the key in Hansen et al., (2017), we determined an individual as *Bertolanius nebulosus*, because there was only a posterior band of teeth in the oral cavity armature (OCA). Moreover, an anterior row of teeth in the posterior band of OCA was not bigger than other teeth in this band and the teeth were not arranged in rows. Also, no cuticular bars by the side of the inner claws were present and lunules on the fourth pair of legs were with fine teeth on their margins.

***Diphascon pingue* group (n=21)** Determination followed Fontoura & Pilato (2007) and original descriptions of *D*.*rudnickii* and *D*.*puchalskii* (Kaczmarek et al., 2018). Individuals had a wide squat body, sub-ventral mouth, and slightly oval pharynx (pharynx ratio max 1.3). Buccal tube measured 37 % of the whole bucco-pharyngeal tube or more. Stylet supports insertion point was at 56 – 60 % of the bucco-pharyngeal tube. Pharynx contained apophyses, three macroplacoids, small microplacoid and a well-developed septulum. Septulum was smaller than the first macroplacoid. Macroplacoid length was gradually increasing from the first one to the third one. Pt index of the whole macroplacoid row was lower than 50. Claws lacked cuticular bars.

***Diphascon nobilei* group** (n=17) Species in the *Diphascon nobilei* group share the following traits: three macroplacoids and a microplacoid in the pharynx, cuticular bars under the first three pairs of legs and claws on the fourth pair of legs different from those on the first three pairs having a wide basal portion with clearly indented margins. Individual species of this group differ by the dimensions and shape of the claws and length of the buccal tube, pharyngeal tube, and macroplacoids (Pilato et al., 2005). However, our individuals had smaller dimensions than provided in the literature (body length 112 – 186 µm), therefore all the dimensions were bit smaller. Moreover, claws were hard to measure accurately due to the suboptimal position, thus we decided to determine this taxon only to the level of the group of similar species, i.e. *D. nobilei* group. Although we could not measure the claws accurately, the external claws seemed to be around 7 µm on all legs (6.10 - 7.21 µm), a bit longer on the fourth legs. Buccal tube measured 14 – 18 µm, pharyngeal tube measured 20.34 - 23.05 µm. The drop-like structure between the buccal and pharyngeal tube was conspicuous and large (length 2.72 - 4.47 µm, width 1.49 - 1.97 µm). Macroplacoid lengths were 2.11 - 2.60, 2.34 - 2.71, 3.26 - 3.95 µm; respectively) and microplacoid length was 1.19 – 1.41 µm.

***Guidettion* cf. *modestum*** (n=7) Observed individuals had pores on the cuticle and a mean body length of 165 µm. Tardigrades had no eye spots, sub ventral mouth, mean buccal tube width of 0.6 µm (internal), 1.6 µm (external), and pharynx length/width ratio of 2.1-2.4. Pharynx contained small apophyses, three macroplacoids, and no microplacoid or septulum. Macroplacoids were in the shape of elongated rods, with lengths of 3.9, 4.6, and 7.5 µm; respectively. Claws were without lunulae or cuticular bars. *G.carolae* is very similar to *G.modestum* and differs by several differences in morphometry. However, the morphometry in the original literature where these differences have been described was measured in two individuals of *G.carolae* and four individuals of *G.modestum* (Binda et al., 1984; Binda & Pilato, 1969), which is not enough to observe statistically meaningful difference (Stec et al., 2016), thus it is possible that *G.modestum* a *G.carolae* are one species. We assigned our individuals to *G.modestum*, because the entire macroplacoid row measured 72-81 % of the pharynx length, while in *G*.*carolae* it should be 63 %. On the other hand, pharyngeal apophyses should be lacking in this species and they were present in our individuals.

***Adropion belgicae*** (n=5) Of all *Adropion* spp., only *A.belgicae* has two very thin macroplacoids and a microplacoid in the elongated pharynx, thus it could not be mistaken for any other *Adropion* species. However, it might be mistaken for *Mesocrista* *spitzbergense* or *M. revelata* which have a similar appearance of bucco-pharyngeal apparatus but differ in the dimensions of bucco-pharyngeal tube. *A. belgicae* has buccal tube width of 2.5 µm, while *Mesocrista* spp. have buccal tube width of 4 µm (Gąsiorek et al., 2016). Furthermore, *Diphascon* spp. with a similar pharynx configuration would have a drop-like structure between the buccal and pharyngeal tubes.

***Adropion scoticum*** (n=4) It was identified by a combination of the following traits: a drop-like structure well developed, very elongated pharynx (pharynx ratio >2), small apophyses in the pharynx, three very long and thin macroplacoids and a microplacoid present, cuticular bars on the first three pairs of legs next to the inner claw and between the claws, claws with smooth bases.

***Platicrista angustata*** (n=3) *Platicrista* had been determined based on the key in Marley (2006). The body of the observed individuals was covered with dense granulation. Body length was 284 – 375 µm. Buccal tube width measured 3 - 4 µm (internal), and 5 – 6 µm (external). The pear-shaped pharynx with a length/width ratio of 2.6 contained two long and thin macroplacoids and no microplacoid or septulum. Lengths of the macroplacoids were 8 and 21 µm; respectively. In the only individual with claws in the optimal position, the posterior claw on the fourth pair of legs had a very wide and stumpy basal part that was widening gradually, ending with lunules. The margins of the lunules are somewhat irregular although, not clearly dentate as in *P.horribilis*. Lunules on other claws were also large and wide, the margin seems to be more or less smooth, and compared to the posterior claw on the fourth pair of legs, the basal portion of the claws was not wide and stumpy, but have a tapering before it widened into a lunule. No cuticular bars were observed.

***Mesobiotus* sp**. (n=2) Cuticle was without pores. Around the mouth opening, ten perribuccal lamellae were present. Pharynx contained 3 rounded rods arranged along a curved line and a microplacoid that was clearly close to the third macroplacoid (closer than its length). Both individuals had Y-shaped claws. A common tract of the claw included an internal septum defining the claw distal part. Species can be distinguished based on the morphology of the eggs (Kaczmarek et al., 2018). For this reason, we determined the individuals only to the generic level.

***Paramacrobiotus richtersi*** **group** (n=16) Cuticle was without pores. Around the mouth opening, ten perribuccal lamellae were present. Pharynx contained three 3 rounded rods and a microplacoid positioned clearly further from the macroplacoids (further than its length). Individuals had Y-shaped claws, with lunules. Species can be distinguished based on egg morphology, thus we determined the individuals to the level of the genus (Kaczmarek et al., 2017).

***Diaforobiotus islandicus*** (n=30) Observed individuals were large (body length 381 – 841 µm) with large eye spots, brownish color, porous cuticle, ten perribuccal lamellae, ventral lamina, dorsal thickening of the buccal tube in the conjunction with a large tooth on the internal surface of the tube. Buccal tube width was 2.72 – 5.35 µm (internal), 5.37 – 8.86 µm (external). Pharynx (ratio 1.1-1.3) contained two macroplacoids (first macroplacoid 7.6 – 21.46 µm, second macroplacoid 5.53 – 12.81 µm) and no microplacoid or septulum. Claws had large lunules with large teeth on their margins. The oral cavity armature was without transverse crests and with strong scattered round teeth present posterior to the second band of teeth (Guidetti et al., 2016).
